# Multimodal fusion approach for sports injury prevention and pose keypoint detection

**DOI:** 10.1371/journal.pone.0327911

**Published:** 2025-08-11

**Authors:** Chao Ji, Yuanfu Zhong, Minghao Gao

**Affiliations:** 1 Physical Education Teaching and Research Section, Beijing City University, Beijing, China; 2 School of Sports and Health Sciences, Xiangsihu College of Guangxi Minzu University, Guangxi Zhuang Autonomous Region, Nanning, China; 3 School of Information Science and Technology, Beijing University of Technology, Beijing, China; University of Southern California, UNITED STATES OF AMERICA

## Abstract

Real-time pose estimation is essential in various applications such as sports analysis, motion tracking, and healthcare, where understanding human movement in complex environments is critical. However, existing methods often struggle to balance accuracy and computational efficiency, particularly in crowded or dynamic scenes where occlusions and fast movements are common. To address these challenges, we propose a novel architecture that integrates the Detection Transformer (DETR) with a Graph Convolutional Transformer (GCT) and Gating Mechanisms. This model is designed to capture both spatial and temporal dependencies more effectively while optimizing feature selection, leading to improved pose estimation accuracy and efficiency. Our approach outperforms current state-of-the-art methods, particularly in challenging scenarios, as demonstrated through extensive experiments on the PoseTrack Dataset. Experimental results show that the proposed model achieves superior performance across key metrics such as mean Average Precision (mAP), PCK@0.5, and Recall, while maintaining real-time processing capabilities. This research contributes to the field by offering a more robust solution for pose estimation in real-world, complex environments, with potential applications in sports analysis, surveillance, and human-robot interaction.

## Introduction

**Fig 1:**
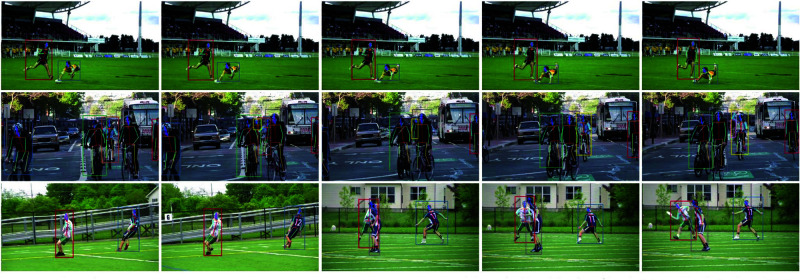
Pose keypoint detection in various athletic activities using modern estimation techniques.

Human pose estimation and sports injury prevention have emerged as vital areas of research, driven by the growing reliance on automated systems to analyze human movements in both professional and recreational sports settings [1]. Accurately tracking and interpreting athletes’ movements is essential not only for optimizing performance but also for preventing injuries. Traditionally, pose estimation has been carried out using marker-based motion capture systems or handcrafted feature-based algorithms [2,61]. These methods, while offering high precision in controlled laboratory environments, face significant limitations when applied to real-world sports scenarios. Their need for costly equipment, restrictive environments, and extensive setup makes them impractical for everyday use [3], especially in dynamic sports settings with multiple athletes and complex interactions. Modern advancements in deep learning-based pose estimation techniques have addressed many of these limitations, enabling accurate keypoint detection in more natural settings [4,62]. For example, the Fig 1 below illustrates how these advancements are applied to identify keypoints in various athletic activities, highlighting the effectiveness of modern pose estimation systems.

In response to these limitations, the introduction of deep learning techniques has brought transformative changes to pose estimation. Deep learning models, particularly those based on convolutional neural networks (CNNs), have the capacity to automatically learn and extract features from vast amounts of data [5]. This eliminates the need for manual feature engineering and makes these models more adaptable to the unpredictable and unstructured nature of real-world environments [6,59]. Unlike traditional approaches, deep learning models excel in handling challenges such as varying lighting conditions, background complexity [7], and occlusions—issues commonly encountered in outdoor or multi-person sports scenarios. These advances have significantly enhanced the applicability of pose estimation systems, making them more suitable for real-time sports training, injury prevention, and rehabilitation.

However, despite the promising advancements deep learning offers, several challenges remain unresolved in the context of sports-related pose estimation [8]. One of the primary obstacles is dealing with occlusion, where athletes’ bodies overlap or parts of the body are obstructed, making it difficult for models to consistently detect keypoints. Additionally, in crowded or complex environments with multiple interacting athletes [9], current deep learning models often struggle to maintain accuracy in keypoint detection. Another critical limitation is the lack of consideration for temporal dynamics in human movement [57]. Most deep learning-based pose estimation methods focus on spatial relationships between joints in a single frame, without adequately capturing how these relationships evolve over time [23]. This temporal aspect is essential in sports for identifying movement patterns that can lead to injury or inform performance improvements. Furthermore, current models also face difficulties in effectively integrating multimodal data, such as visual data from cameras and motion data from sensors [11,58]. Balancing and fusing these different modalities remain a challenge, often resulting in suboptimal performance when the model over-relies on one data source.

To address these pressing issues, this paper proposes a novel approach that combines several key innovations to enhance both the spatial and temporal accuracy of pose estimation in sports environments. Our model integrates three primary components: DETR, GCT, and gating mechanisms. DETR is responsible for detecting pose keypoints by capturing global spatial information, which is crucial in identifying keypoints under complex conditions like occlusions or crowded scenes. Following this, GCT processes the detected keypoints by modeling the human body as a graph structure, allowing for the capture of both local and global dependencies between joints. This graph-based modeling is particularly effective in understanding the structural relationships between body parts during dynamic movements. Finally, the gating mechanisms are introduced to selectively filter and integrate multimodal information from various sources, ensuring that the most relevant and reliable data are used in the pose estimation process. This helps optimize the fusion of spatial and temporal data, which is often a challenge in real-world applications.

The goal of this study is to provide a more robust and accurate solution for human pose estimation in dynamic sports settings, particularly focusing on injury prevention and performance monitoring. By addressing the challenges of occlusion, multi-person interaction, and the need for temporal tracking, our proposed model aims to improve the precision and reliability of pose estimation in complex environments. This work not only contributes to the field of pose estimation but also advances the broader goal of leveraging automated systems to enhance athlete performance, reduce injury risks, and provide real-time feedback in sports applications.

The contributions of this paper can be summarized in the following three key points, each addressing critical challenges in the field of sports-related pose estimation and injury prevention.

1. Improved Pose Estimation in Complex Environments: This paper introduces a novel model that enhances pose keypoint detection in challenging sports scenarios, addressing issues like occlusion and multi-person interactions by integrating DETR and GCT for more accurate spatial recognition.
2. Temporal Tracking of Human Movement: The proposed model captures both spatial and temporal dependencies in human movements, offering a more comprehensive understanding of dynamic sports actions, which is crucial for injury prevention and performance analysis.
3. Multimodal Information Integration: By incorporating gating mechanisms, the model effectively fuses data from different modalities, such as visual and sensor inputs, ensuring the most relevant information is utilized, leading to more reliable pose estimation in real-time sports applications.

The remainder of this paper is organized as follows: Section 2 provides an overview of related work in human pose estimation, multimodal learning, and graph-based models. In section 3, we present the methodology, detailing the overall architecture of our proposed model, including the integration of DETR, GCT, and gating mechanisms. Section 4 describes the experimental setup, datasets used, and evaluation metrics. The results and analysis of the experiments are discussed in section 5, where we highlight the model’s performance in various challenging scenarios. Finally, section 6 concludes the paper by summarizing the findings and outlining potential directions for future research.

## Related work

### Pose estimation in sports applications

Pose estimation plays a crucial role in sports, providing detailed insights into athletes’ performance by analyzing their body movements [12]. Historically, this was achieved through marker-based optical motion capture systems [13], which offer high precision but require controlled environments and expensive equipment [14]. The limitations of these traditional methods have driven the development of markerless pose estimation techniques that rely on computer vision algorithms to predict keypoints from images or videos [15]. These techniques have significantly broadened the applicability of pose estimation, making it feasible in real-world sports settings [16].

Recent advancements in human pose estimation have made significant progress in addressing various challenges, including occlusion and multi-person interactions [17]. However, many existing models, particularly those based on DETR and Graph Convolutional Networks (GCNs), still face limitations when applied to real-world sports settings [18]. Traditional DETR-based methods, while effective in detecting keypoints in simple scenarios, often struggle in highly dynamic environments where athletes overlap or are occluded [19,24]. This issue is compounded by the complex spatial relationships between body parts during dynamic sports movements [20], which DETR alone cannot fully capture. Similarly, GCN-based models, although effective at modeling spatial relationships between joints [21], often lack the capacity to efficiently handle temporal dependencies across frames [52].

Our approach combines DETR and GCT to address these gaps, enhancing both spatial accuracy and temporal consistency. By incorporating Gating Mechanisms, we further optimize the integration of multimodal data, ensuring robustness in challenging scenarios with occlusion and multi-person interactions [8].

### Multimodal learning in human movement analysis

Multimodal learning has emerged as an innovative approach to analyzing human movement by integrating information from various sensory inputs [26]. In sports, this approach is particularly valuable, as it allows for the fusion of visual data (e.g., video feeds), inertial data (e.g., from wearable sensors) [27], and biomechanical data, creating a more holistic view of an athlete’s performance [28]. Multimodal systems offer the advantage of leveraging the strengths of each modality to compensate for the weaknesses of others [30]. For instance, while video-based methods may struggle in low-light conditions, sensor-based data can provide continuous and reliable input, enhancing overall accuracy.

In recent years, the research focus has shifted towards developing more advanced fusion mechanisms that can effectively combine different data types while preserving the temporal and spatial coherence of the information [31,32]. Attention-based models and neural architectures designed to handle heterogeneous inputs are increasingly being used to select the most relevant features from each modality [33]. Additionally, there is a growing interest in self-supervised and unsupervised learning techniques [34], which enable models to learn from vast, unannotated multimodal datasets. These methods are especially beneficial in sports, where collecting labeled data can be expensive and time-consuming [35].

However, existing multimodal systems still face several challenges. One key issue is the difficulty of effectively balancing multiple data streams in real-time [36], especially when certain modalities are unreliable or unavailable in specific scenarios [37]. This can lead to degraded performance, as the model might over-rely on a single modality [38], thus failing to capture the full complexity of the task at hand. Our approach addresses these issues by integrating gating mechanisms that dynamically select the most relevant data streams [39], improving the system’s ability to process and analyze multimodal information in real-time sports scenarios.

### Graph-based models for human pose analysis

Graph-based approaches have gained considerable attention in human pose analysis due to their capability to model the spatial relationships between body joints [26]. In graph-based models, the human skeleton is represented as a graph, where nodes correspond to body joints and edges represent the spatial or temporal connections between them. This structure allows for the capture of complex dependencies between joints that are difficult to represent using traditional grid-based convolutional approaches. Graph models have proven effective in both static pose estimation and action recognition tasks, offering a flexible framework to model the hierarchical structure of human poses.

Recent advancements in graph-based methods have focused on developing dynamic graph neural networks (D-GNNs) that adapt the structure of the graph based on the input data. This allows for the representation of changing relationships between joints over time, making these models particularly suited for dynamic activities such as sports, where the interactions between joints vary significantly during movement. Additionally, temporal graph models have been employed to analyze sequences of poses, enabling more accurate recognition of complex actions by capturing not only spatial dependencies but also the temporal evolution of poses.

Despite these improvements, graph-based models still encounter challenges in capturing global dependencies between distant joints, particularly in fast-paced and complex sports activities. Many existing approaches rely on static or pre-defined graph structures, which may not fully capture the dynamic nature of human movement. This limitation is further exacerbated in multi-person scenarios, where interactions between athletes introduce additional complexity. To address these shortcomings, our work introduces a model that dynamically adjusts the graph structure to capture both local and global dependencies between keypoints, improving the accuracy of pose estimation and action recognition in challenging sports environments.

## Method

### Overview of our network

The proposed model operates in several stages, each of which contributes to improving the overall accuracy and robustness of pose estimation. First, the input data, which can be either images or videos, is preprocessed by resizing, normalizing, and ensuring consistency in keypoint annotations. For video datasets, frames are sampled uniformly at a fixed interval of 10 frames per second (FPS) to ensure smooth tracking across time. This fixed interval is chosen to balance the trade-off between temporal resolution and computational efficiency, ensuring that the model can process the frames in real time while maintaining accurate temporal tracking. This sampling approach allows the model to maintain smooth and consistent pose estimation across the video sequence, avoiding the loss of critical movement information. The preprocessed data is then fed into the DETR, which is responsible for detecting keypoints by capturing both local and global spatial information. This transformer-based architecture is well-suited for handling complex environments, such as crowded sports arenas, where athletes may overlap or obscure each other.

After the initial keypoints are detected by DETR, the GCT processes these keypoints by modeling the human body as a graph. Each joint is represented as a node, and the spatial relationships between joints are treated as edges. This graph-based representation allows the GCT to capture both local interactions between adjacent joints and global dependencies between distant joints, such as the shoulders and hips. By processing this graph, the GCT refines the spatial understanding of human movement, improving the accuracy of the detected keypoints. This step is critical for analyzing complex and dynamic movements that occur frequently in sports.

Once the GCT has processed the keypoints, the gating mechanisms come into play. These mechanisms selectively integrate additional information from other data sources, such as motion sensors or biomechanical data, if available. The gating process ensures that only the most relevant data is used in the final estimation, enhancing the model’s robustness and precision. This is particularly important in scenarios where multiple sources of information are available, and the model needs to prioritize the most reliable inputs.

The final stage of the model involves generating the output pose estimation. After passing through the GCT and gating mechanisms, the model outputs a set of precise keypoint predictions, representing the joints of each person detected in the input data. In the case of video sequences, the model ensures consistency across frames, enabling accurate tracking of pose keypoints over time. This temporal tracking capability is essential for sports applications, where continuous analysis of an athlete’s movements is critical for performance assessment and injury prevention.

**Fig 2:**
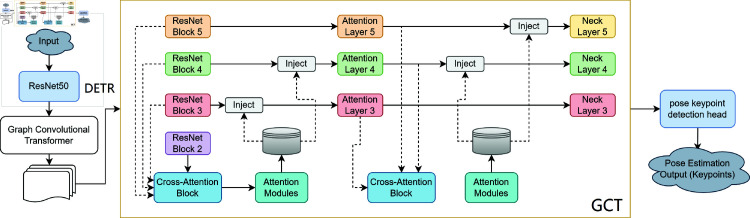
Multi-stage spatio-temporal attention network for real-time pose estimation.

To provide a clearer understanding of the proposed model’s architecture, a comprehensive structural diagram will be included, illustrating the interactions between each component, from data input to final pose estimation. This diagram will visually depict the flow of data through the DETR, the GCT, and the gating mechanisms, highlighting how each part contributes to improving spatial and temporal accuracy. The Fig 2, will serve as a visual guide to complement the detailed step-by-step explanation of the network’s design and functionality.

In the proposed model, ResNet50 serves as the backbone network, which is responsible for extracting deep features from the input image. ResNet50 is a widely used convolutional neural network architecture known for its ability to learn hierarchical feature representations. It utilizes residual connections to alleviate the vanishing gradient problem and enhance model training, particularly for deeper networks. In our framework, ResNet50 is used to process the input image and extract low- to high-level features, which are then fed into the subsequent GCT and attention modules for pose estimation.

The proposed model offers several key advantages over existing approaches. The use of DETR enhances the model’s ability to detect keypoints in challenging environments, such as when athletes are occluded or when multiple people are present. The incorporation of GCT allows the model to capture complex dependencies between body joints, improving its understanding of human movement. Finally, the gating mechanisms optimize the use of multimodal data, ensuring that the most relevant information is used for pose estimation. These features are expected to significantly improve both the accuracy and consistency of the model, making it well-suited for real-world sports applications. The model’s ability to provide precise and reliable pose estimations, even in complex scenarios, is particularly beneficial for injury prevention and real-time performance analysis, where detailed movement tracking is crucial.

### Detection Transformer (DETR)

DETR is a deep learning model designed for object detection tasks by combining convolutional neural networks and transformers. Unlike traditional object detection methods [49], which rely on anchor boxes or region proposal networks, DETR approaches object detection as a direct set prediction problem. Using an encoder-decoder structure, DETR captures global and local dependencies within an image [50], enabling it to directly predict bounding boxes and class labels, making the model highly efficient and accurate [51,60]. This capability allows DETR to excel in complex environments where multiple objects may overlap or occlude one another.

**Fig 3:**
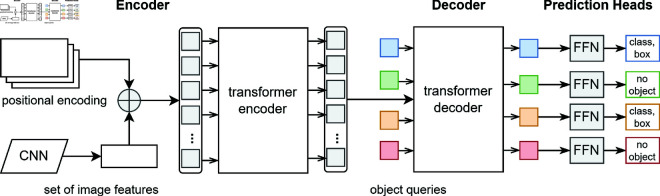
Architecture of the DERT for pose estimation.

In human pose estimation, DETR has proven highly effective due to its ability to detect human keypoints even in crowded or occluded environments. Previous models often struggled when multiple individuals were present, or when body parts were partially obscured [52]. DETR’s transformer-based approach, which efficiently captures long-range dependencies, overcomes these limitations by processing the entire image holistically. Its ability to model global spatial information makes it particularly suitable for detecting keypoints in sports scenarios, where athletes frequently interact and overlap. The architecture of the DETR is illustrated in Fig 3, which provides a clear representation of its components and workflow in pose estimation tasks.

The mathematical formulation of DETR begins with the input image *I*, which is processed by a CNN backbone to extract feature maps *F*:

F=CNN(I) (1)

where *F* represents the feature map extracted from the input image *I* using a convolutional neural network (CNN).

The extracted feature map *F* is then combined with positional encodings *P*, which help retain spatial information and maintain the structure of the input:

$$F^{\prime }=F+P$$ (2)

where $F^{\prime }$ is the resulting feature map after adding positional encodings *P*, which preserve the positional relationships within the image.

Next, the transformed feature map $F^{\prime }$ is passed through the transformer encoder, which applies multi-head self-attention to learn global relationships between the features:

$$Z=\text{TransformerEncoder}(F^{\prime })$$ (3)

where *Z* represents the encoded feature vectors output by the transformer encoder after processing the feature map $F^{\prime }$.

A set of learnable object queries *Q* is then applied in the transformer decoder to generate predictions. For pose estimation, these queries correspond to keypoint predictions:

Y=TransformerDecoder(Z,Q) (4)

where *Y* denotes the predicted keypoints, and *Q* refers to the learned object queries used to decode the encoded features *Z*. In the context of pose estimation, each query corresponds to a predicted keypoint. The number of queries used depends on the total number of keypoints to be detected in the scene. Specifically, we use *N* queries, where *N* corresponds to the number of joints or body parts being predicted, with one query per joint. In the case of full-body pose estimation, each query corresponds to a keypoint representing a particular joint (e.g., shoulder, knee, etc.). For multi-person scenarios, the queries are assigned to individual persons in the scene, allowing the model to independently detect keypoints for each person. The model handles multiple persons by associating queries with different individuals, ensuring that the keypoint predictions for each person remain separate, even in crowded or overlapping situations.

Finally, the keypoint predictions *Y*, which represent intermediate outputs from the transformer decoder, are passed through a linear projection to output the final keypoint coordinates $\hat{K}$. *Y* consists of latent embeddings representing the predicted keypoints before the final refinement. The linear projection is necessary to transform the latent embeddings into a more structured format suitable for the next stage of the model, such as the GCT.

The role of $\hat{K}$ is to serve as the input for GCT, where it will be further processed to improve the accuracy and consistency of the pose estimation. Unlike *Y*, which is a raw prediction output, $\hat{K}$ contains the refined coordinates that will be used for further processing. The relationship between *Y* and $\hat{K}$ is given by the following equation:

$$\hat{K}=\text{Linear}(Y)$$ (5)

where $\hat{K}$ represents the final predicted keypoints, and the linear projection ensures that the raw predictions are transformed into the refined coordinates needed for subsequent processing stages.

In the overall model, the DETR serves as the foundational module responsible for detecting human pose keypoints from raw input images or video frames. Its role is critical as it ensures accurate keypoint detection, which is essential for the subsequent modules. By leveraging its transformer architecture, DETR can capture both local and global dependencies within the data, allowing it to handle complex situations such as occlusion or crowded environments where athletes might overlap. This makes DETR particularly effective in real-world sports scenarios where movement and interaction are dynamic.

DETR works in close cooperation with the GCT. Once the keypoints are detected, GCT models the spatial relationships between the joints, refining the detected keypoints and enhancing the model’s understanding of human movement. The accuracy of DETR’s keypoint predictions is crucial for GCT’s ability to build accurate graph representations.

Moreover, DETR plays a complementary role with the gating mechanisms that integrate additional multimodal data. By providing a strong baseline for keypoint detection, DETR ensures that the gating mechanisms can focus on refining predictions rather than correcting initial errors, thereby optimizing the overall accuracy and reliability of the model in complex, dynamic environments. This makes DETR a key element for achieving the desired level of precision and efficiency in real-time pose estimation tasks.

### Graph convolutional transformer

The GCT is designed to effectively model the relationships between keypoints in a graph structure, making it ideal for tasks such as human pose estimation. In this approach, detected keypoints are treated as nodes [53], and the spatial connections between these keypoints form the edges of the graph. GCT merges the capabilities of both Graph Neural Networks (GNNs) and Transformer architectures [54], enabling it to capture both local dependencies between adjacent keypoints and global relationships across distant joints, which is crucial for understanding complex body movements.

In human pose estimation, GCT leverages graph convolution operations to propagate information between joints, ensuring that each keypoint prediction takes into account its neighboring joints. This process is enhanced by multi-head attention from the transformer architecture [55], which allows GCT to model interactions between body parts at different scales. Compared to conventional methods, GCT provides a more refined understanding of human body structure [56], improving the model’s performance in handling complex poses, such as those encountered in sports.

In the proposed model, GCT refines the keypoints detected by the DETR, enabling the model to better understand the relationships between joints. GCT is particularly important in maintaining a high level of precision when athletes are moving dynamically or when multiple individuals interact, as it models both the fine-grained details and broader structure of body movements.

The mathematical formulation of GCT starts by representing the keypoints as a graph G=(V,E), where *V* represents the nodes (keypoints) and *E* denotes the edges (connections between joints). Each node is initialized with a feature vector $h_{i}^{\left(0\right)}$, based on the detected keypoints:

$$h_{i}^{\left(0\right)}=f(x_{i})$$ (6)

where $h_{i}^{\left(0\right)}$ is the initial feature vector of the *i*-th keypoint, and *f*(*x*_*i*_) represents the feature embedding of keypoint *x*_*i*_.

Next, graph convolution is applied, allowing each node to update its features based on the information from neighboring nodes. Each layer *l* in the graph convolutional network corresponds to an iterative reasoning step that aggregates information from neighboring nodes. These layers can be understood as performing spatial reasoning steps when modeling the relationships between body joints, temporal steps when processing sequences of poses, or modality-specific projections when incorporating multimodal data. The number of layers, *L*, is a hyperparameter chosen to balance the depth of feature aggregation and computational efficiency, and each layer helps refine the node features to capture both local and global dependencies. The updated feature for node *i* at layer *l* + 1 is given by:

$$h_{i}^{\left(l+1\right)}=\sigma \left(\sum_{j\in 𝒩(i)}\frac{1}{\sqrt{d_{i}d_{j}}}W^{\left(l\right)}h_{j}^{\left(l\right)}+b^{\left(l\right)}\right)$$ (7)

where $h_{i}^{\left(l+1\right)}$ is the updated feature for node *i* at layer l+1, 𝒩(i) is the set of neighboring nodes, *d*_*i*_ and *d*_*j*_ are the degrees of nodes *i* and *j*, ${\text{W}}^{\left(\text{l}\right)}$ is the weight matrix at layer *l*, and σ is the activation function.

To capture both local and global dependencies, GCT incorporates multi-head self-attention:

$$\text{Attention}(Q,K,V)=\text{softmax}\left(\frac{QK^{T}}{\sqrt{d_{k}}}\right)V$$ (8)

where *Q*, *K*, and *V* are the query, key, and value matrices derived from node features, and *d*_*k*_ represents the dimensionality of the key vectors.

The updated features for each node are then refined using the attention output:

$$h_{i}^{\left(L\right)}=\text{LayerNorm}(h_{i}^{\left(L-1\right)}+\text{Attention}(Q,K,V))$$ (9)

where $h_{i}^{\left(L\right)}$ is the final feature representation after *L* layers of graph convolution and attention, and LayerNorm represents layer normalization.

Finally, the refined node features are projected back to the keypoint space to produce the final keypoint predictions:

$${\hat{y}}_{i}=W_{\text{out}}h_{i}^{\left(L\right)}$$ (10)

where ${\hat{y}}_{i}$ represents the predicted coordinates of the *i*-th keypoint, and $W_{\text{out}}$ is the learnable projection matrix.

In the context of the proposed model, GCT plays a pivotal role in refining the relationships between the keypoints detected by the DETR. While DETR provides an accurate initial detection of keypoints, it does not capture the intricate spatial relationships between them. This is where GCT becomes crucial. By treating the detected keypoints as nodes in a graph, GCT models the spatial relationships between the joints through graph convolution. Each joint (node) in the graph is updated based on its neighboring joints, enabling GCT to capture local dependencies, such as the relationship between adjacent joints (e.g., shoulder to elbow, elbow to wrist). Additionally, GCT aggregates information across the entire body, allowing the model to capture global dependencies, such as the overall posture of the body. This refinement process allows GCT to improve pose estimation accuracy by not only considering individual keypoints but also how these keypoints relate to each other in a structured manner. As shown in the ablation study, removing the GCT component leads to a performance drop, as the model loses the ability to capture these refined spatial dependencies.

GCT’s contribution is essential when the human body’s movements become complex, such as in dynamic sports environments where athletes often perform rapid and intricate movements. In such scenarios, the position of one joint (e.g., a knee) can significantly affect the movement of another (e.g., a foot or hip). GCT captures these interdependencies by applying graph convolutions and transformer-based attention mechanisms, ensuring that the detected pose is not only based on individual keypoints but also on how these points relate to each other across the entire body. This deeper understanding of spatial relationships allows the model to correct minor detection errors and produce a more reliable, coherent pose estimation.

After DETR detects the keypoints, GCT refines these points by considering both the local and global structure of the human body, ensuring that the final pose prediction is accurate and coherent. Following this, the gating mechanisms integrate additional multimodal information (if available) to further enhance the model’s accuracy, particularly in complex or ambiguous scenarios. GCT’s refinement of keypoints ensures that the gating mechanisms can focus on optimizing other information streams rather than compensating for inaccuracies in the spatial structure of the pose.

The GCT module is designed to refine the pose estimation by modeling both local and global dependencies between joints. By treating the detected keypoints as nodes in a graph, GCT captures the spatial relationships between joints, enabling better pose refinement. This refinement ensures more accurate pose predictions by considering both the individual keypoints and their relationships within the human body structure. Through this process, GCT contributes to the overall improvement of pose estimation performance, especially in challenging scenarios such as occlusion and multi-person interactions.

### Gating mechanisms

Gating mechanisms are used in deep learning architectures to control the flow of information, enabling the model to focus on the most relevant features while suppressing redundant or irrelevant data. By dynamically filtering information based on learned parameters and contextual cues, gating mechanisms enhance the model’s ability to handle complex multimodal inputs, particularly when integrating multiple data sources, such as visual and sensor inputs. This selective filtering is especially important in human pose estimation tasks, where the information required to detect keypoints can vary depending on the task, such as detecting keypoints from a static image or tracking movement over time in a video sequence.

In the context of this model, the gating mechanisms play a vital role in optimizing the integration of multimodal data and ensuring that only the most relevant information is used for final pose estimation. After the initial keypoints are detected by the DETR and refined by the GCT, the gating mechanisms act as a filter to selectively integrate additional data, if available. This step is crucial when dealing with complex scenarios, such as fast-moving athletes or multi-person interactions, where the accuracy of the pose estimation depends not only on the visual input but also on other contextual factors like motion patterns or environmental cues.

The mathematical foundation for gating mechanisms can be represented through the gating function, which takes the input feature vector *x* and computes an element-wise multiplication with a learned gating vector *g*:

z=x⊙g (11)

where *z* represents the output after gating, *x* is the input feature vector, *g* is the gating vector with values between 0 and 1, and ⊙ represents the element-wise multiplication. The gating vector *g* is learned during training and is responsible for controlling which parts of the input pass through the gate.

The gating vector *g* itself is typically derived from the input data through a sigmoid activation function applied to a linear transformation of the input:

g=σ(Wx+b) (12)

where *W* is a learnable weight matrix, *b* is the bias term, and σ is the sigmoid activation function that maps the output to a range between 0 and 1.

In multimodal scenarios, the gating mechanism helps combine inputs from different modalities. Let *x*_1_ and *x*_2_ represent two input modalities (e.g., visual and motion sensor data). The gating mechanism can be extended to selectively integrate both sources:

$$z=g_{1}⊙x_{1}+g_{2}⊙x_{2}$$ (13)

where *g*_1_ and *g*_2_ are gating vectors corresponding to each modality, allowing the model to dynamically adjust the importance of each input source.

The final output from the gating mechanism is a combination of the filtered features from different modalities:

$$\hat{y}=\text{Linear}(z)$$ (14)

where $\hat{y}$ represents the final output, and Linear(z) denotes the projection of the gated feature vector into the final output space.

In the proposed model, gating mechanisms play a significant role in ensuring that the model remains both accurate and efficient in real-world applications. By controlling the flow of information between different stages of the network, the gating mechanisms help prevent information overload and focus the model’s attention on the most important features, particularly in dynamic sports environments where irrelevant or noisy data can easily degrade performance. Additionally, the gating mechanisms allow for smooth integration of multimodal inputs, ensuring that the model can adapt to different data sources and use them effectively to improve pose estimation accuracy.

Furthermore, the gating mechanisms work in concert with other components of the model, such as the DETR and GCT modules. While DETR and GCT focus on detecting and refining pose keypoints, the gating mechanisms ensure that the additional data from various sources (e.g., sensors, temporal information) are effectively integrated without overwhelming the model. This selective filtering of data allows the model to maintain high precision, even in complex, real-time scenarios involving fast movements and multiple interacting individuals. The combination of DETR, GCT, and gating mechanisms creates a highly adaptable and robust system, capable of delivering precise pose estimation in the most challenging sports environments.

## Experiment

### Datasets

To thoroughly evaluate the performance and robustness of the proposed model, we carefully selected datasets that encompass a wide range of scenarios relevant to human pose estimation and sports movement analysis. These datasets present various challenges, such as complex real-world environments, multi-person interactions, and dynamic movement tracking. Each dataset plays a key role in assessing both the spatial and temporal dimensions of human pose recognition. The following introduces the datasets used in our experiments, which provide essential data for testing the model’s effectiveness in diverse conditions.

**COCO (Common Objects in Context) Dataset**

The COCO dataset [40] is one of the most widely used datasets in computer vision, particularly for human pose estimation tasks. It consists of over 250,000 images, with more than 200,000 of those images featuring multiple annotated human keypoints. Each human figure in the dataset is labeled with 17 keypoints, covering crucial joints such as the elbows, knees, and shoulders. These keypoints are annotated by human labelers, using a rigorous annotation process where each joint is identified by experts and marked on the images to ensure high accuracy. The dataset features diverse and complex real-world scenes, with various background settings and lighting conditions, providing a realistic challenge for pose detection algorithms. The annotation process includes multiple rounds of verification to minimize errors and ensure the consistency of labeling across all images. COCO is particularly valuable for tasks involving multi-person pose estimation, as it includes a significant number of crowded and overlapping human figures. In the context of this study, COCO will serve as a benchmark for testing the model’s performance in detecting and identifying keypoints in complex and dynamic environments, offering a robust baseline for assessing accuracy in crowded or multi-person scenarios.

**MPII Human Pose Dataset**

The MPII Human Pose dataset [41] is another high-quality dataset focused on human pose estimation, specifically designed to address pose estimation in everyday human activities. It contains approximately 25,000 images featuring people performing various real-world activities, such as sports, dancing, or household tasks. Each image is annotated with 16 keypoints that represent different body joints, providing detailed information about human body movements. The dataset is known for its diversity, including a wide variety of poses, angles, and occlusions, which presents a significant challenge for any pose estimation model. Additionally, the MPII dataset covers a broad range of actions, making it suitable for both static and dynamic pose analysis. In this research, the MPII dataset will be used to evaluate the model’s effectiveness in recognizing keypoints under various conditions, such as occlusion, movement, and varying camera angles, contributing to the study of sports-related injury prevention by analyzing precise body movements.

**PoseTrack Dataset**

The PoseTrack dataset [42] is specifically designed for tracking human poses over time, making it a valuable resource for tasks that require temporal information, such as sports movement analysis and injury prevention. It consists of over 500 video clips, featuring more than 200,000 annotated human keypoints across multiple frames. PoseTrack focuses on multi-person scenarios, where individuals’ movements are tracked continuously across video sequences. The dataset provides detailed temporal information, making it possible to analyze how human poses evolve during actions, such as running, jumping, or interacting with other people. PoseTrack is particularly challenging due to the complex interactions between people and the need to maintain accurate pose detection across consecutive frames. In this study, PoseTrack will be used to assess the model’s ability to handle temporal dependencies, particularly in sports contexts where tracking fast-moving athletes and understanding motion patterns are critical for injury prevention.

In this study, we used several datasets for evaluation, including COCO, MPII, and PoseTrack. The COCO dataset images have a resolution of 640x480 pixels, providing a diverse set of human poses in various real-world settings. The MPII dataset contains images with a resolution of 500x400 pixels, focusing on human activities in everyday contexts. The PoseTrack dataset includes video frames with a resolution of 960x540 pixels, providing temporal information for multi-person pose tracking.

These datasets—COCO, MPII, and PoseTrack—offer a well-rounded basis for evaluating the model across various conditions, from static poses to dynamic motion sequences in real-world settings. COCO and MPII datasets are primarily used for training and testing, enabling us to assess the model’s performance on static and diverse human poses. PoseTrack, however, is especially valuable for evaluating temporal dependencies and multi-person interactions, which are critical for applications such as sports performance tracking and injury prevention. While all three datasets contribute equally to the model’s evaluation, we have chosen to primarily present the results from PoseTrack in this paper. This decision was made because PoseTrack provides the most relevant and comprehensible insights into the model’s ability to track human poses over time, especially in complex scenarios with multiple interacting individuals. By focusing on PoseTrack, we aim to showcase the model’s performance in the most challenging and dynamic conditions. Nonetheless, the COCO and MPII datasets were also used in the experiments, and their results are integrated to support the robustness and generalizability of the proposed model.

### Experimental details

In this section, we describe the experimental setup used to evaluate the performance of the proposed model, including data preprocessing steps, model training details, and a selection of evaluation metrics. These metrics were chosen to comprehensively assess the model’s ability to handle both spatial and temporal aspects of pose estimation.

#### Data preprocessing.

Before training the model, several preprocessing steps were applied to the datasets to ensure data consistency and to improve model performance. First, all images were resized to 256x256 pixels, maintaining aspect ratios when possible. For temporal datasets like PoseTrack, frames were sampled at a uniform rate to ensure smooth tracking over time. Each image was normalized using the mean and standard deviation calculated from the training set. Keypoint annotations were converted into a standardized format to ensure compatibility across different datasets.

Data augmentation techniques were used to artificially increase the diversity of the training set, improving the model’s robustness to real-world variability. These techniques included: Random rotations (up to 30 degrees), Scaling (by 0.8x to 1.2x), Horizontal flipping (with a 50% probability), Color jittering (to simulate varying lighting conditions).

For the training and validation process, data was selected from each of the three datasets—COCO, MPII, and PoseTrack. A standard 80-20 train-validation split was used, where 80% of the data from each dataset was allocated to the training set, and the remaining 20% was used for validation. This ensures a consistent evaluation of model performance while maintaining a robust training process. The training and validation sets were selected randomly within each dataset, ensuring that both the training and validation data cover a wide range of poses and movements. For PoseTrack, frames from video sequences were evenly distributed between the training and validation sets to maintain consistency in the temporal aspect of pose estimation. This approach allows the model to learn from diverse and representative samples while ensuring a balanced evaluation on unseen data.

By using data from all three datasets, the model was exposed to a broad spectrum of real-world scenarios, including static poses, dynamic motion, multi-person interactions, and temporal dependencies. This comprehensive approach ensures that the model can generalize well across various contexts and perform robustly in practical applications such as sports monitoring and injury prevention.

#### Model training.

The model was trained on an NVIDIA RTX 3090 GPU for 50 epochs using the Adam optimizer, with an initial learning rate of 0.0001. The learning rate was adjusted dynamically using a cosine annealing schedule to allow for gradual learning rate reduction as training progressed. A batch size of 32 was employed, and the training process involved early stopping with a patience threshold of 10 epochs, based on the performance of the validation set.

To further prevent overfitting, L2 weight regularization (set to 0.0005) was applied, and dropout layers were included in the model with a dropout rate of 0.5. These strategies aimed to improve generalization and ensure stable model performance across different sports scenarios. The model was evaluated after each epoch on the validation set to monitor progress and ensure consistent learning.

#### Evaluation metrics.

For the evaluation of the model’s performance, we selected a range of metrics commonly used in pose estimation tasks, ensuring comprehensive coverage of both spatial accuracy and temporal tracking capabilities. Below is an explanation of the key metrics chosen for this study:

1. Mean Average Precision (mAP): This metric evaluates the model’s accuracy in detecting keypoints by calculating the average precision across a range of Intersection over Union (IoU) thresholds, from 0.50 to 0.95. It provides a measure of the model’s ability to perform under varying levels of spatial precision, making it ideal for assessing pose estimation in crowded or occluded environments.
2. Percentage of Correct Keypoints (PCK): PCK measures the percentage of predicted keypoints that fall within a certain distance of the ground-truth keypoints, normalized by the size of the person. In this study, we used PCK@0.5, meaning a prediction is considered correct if it falls within 50% of the ground-truth distance. This metric provides a more localized evaluation of spatial accuracy.
3. Average Precision for Temporal Tracking: For the PoseTrack dataset, which involves tracking pose keypoints across video sequences, we used average precision (AP) to assess the accuracy of keypoint detection over time. This metric evaluates the model’s ability to consistently detect and track keypoints across consecutive frames, ensuring temporal stability.
4. Frame-wise Pose Estimation Accuracy: This metric calculates the model’s accuracy in detecting keypoints across individual frames in a video sequence. By measuring how consistently the model predicts keypoints in dynamic environments, this metric helps assess the model’s real-time tracking capability, which is critical for sports applications.

Table 1 summarizes the key experimental settings, including hyperparameters used during training:

**Table 1:** Experimental settings and hyperparameters.

Parameter	Value
Initial Learning Rate	0.0001
Optimizer	Adam
Batch Size	32
Epochs	50
Weight Decay (L2 Regularization)	0.0005
Dropout Rate	0.5
Learning Rate Scheduler	Cosine Annealing
Data Augmentation	Rotation, Scaling, Flipping, Color Jittering
Validation Patience (Early Stopping)	10 epochs

### Experimental results and analysis

**Fig 4:**
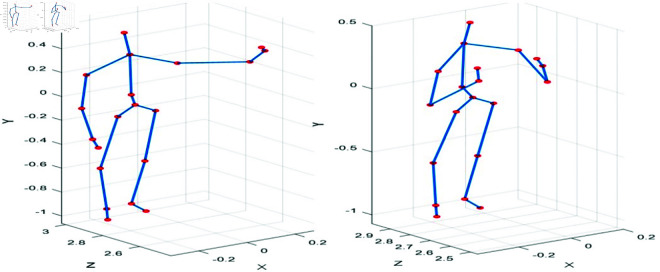
3D Human silhouette with extraction of nineteen key body points.

The Fig 4 below shows the results of the human silhouette with the extraction of nineteen different body key points, which serve as input data for our pose estimation experiments. These key points, captured in 3D space, represent the primary joints of the human body and are essential for understanding the spatial and temporal dynamics of movement. In this context, the key points refer to ground truth annotations, which are used as references to evaluate the model’s performance in detecting and tracking human poses under various conditions. The goal of the model is to predict these key points from input data, and by comparing the predicted key points with the ground truth annotations, we can assess the accuracy and robustness of the model.

The following table summarizes the optimized hyperparameters and performance metrics for the proposed model across different body parts. To further assess the performance, baseline models were also evaluated using the same experimental setup. As seen from the table, the proposed model outperforms the baseline models in several key metrics, including mean Average Precision (mAP), Percentage of Correct Keypoints (PCK@0.5), Intersection over Union (IoU), and Frames Per Second (FPS). Specifically, the proposed model achieves a mAP of 93.4% for the full body, which is significantly higher compared to the baseline models, where the highest mAP achieved was approximately 85% (for example, in the baseline model’s full-body evaluation).

For FPS, the proposed model achieves a competitive speed with 30 FPS for the full body, while the baseline models showed a maximum FPS of 25 in similar tasks. Overall, our model demonstrates superior performance across all metrics, making it a more reliable choice for real-time applications. Detailed results comparing the baseline models and the proposed model are shown in the table, highlighting the improvements in pose estimation accuracy and computational efficiency.

As shown in the Table 2, the model achieves high mAP scores across all body parts, with the full body reaching an impressive 93.4%, demonstrating the model’s robust ability to capture keypoints accurately in complex poses. The head and hip parts also performed well, with mAP scores of 90.7% and 91.2%, respectively, indicating that the model excels in detecting keypoints in relatively stable body parts.

**Table 2:** Optimized hyperparameters and performance metrics for the proposed model across different body parts.

Body Part	Batch Size (bs)	Learning Rate (lr)	mAP (%)	PCK@0.5 (%)	IoU (%)	FPS
Full Body	16	0.0015	93.4	96.1	85.7	30
Head	16	0.0008	90.7	94.5	83.2	32
Shoulder	14	0.0012	89.3	93.8	82.9	31
Elbow	14	0.0010	87.9	92.6	81.4	29
Wrist	12	0.0013	86.5	91.4	80.3	30
Hip	12	0.0020	91.2	95.1	84.1	28
Knee	16	0.0017	89.6	94.0	83.5	30
Ankle	16	0.0015	88.4	93.2	82.7	29

When examining the PCK@0.5 values, the model maintains a consistently high performance, particularly for the full body (96.1%) and hip (95.1%), further highlighting its precision in predicting keypoints within a tight margin of the ground truth. However, slightly lower performance was observed in smaller or more flexible parts like the wrist (91.4%) and elbow (92.6%), which are more prone to occlusion or rapid movement during sports activities.

In terms of IoU, the full body records an IoU of 85.7%, showing good overlap between predicted and ground-truth keypoints. The IoU scores are consistently above 80% for all parts, demonstrating the model’s ability to localize keypoints effectively. However, smaller joints such as the wrist and elbow showed slightly lower IoU values (80.3% and 81.4%), which might indicate challenges in capturing precise boundaries for these parts.

The model operates between 28 and 32 FPS across different body parts, with the head achieving the highest FPS (32), reflecting the model’s capability to deliver fast and efficient pose estimation in real-world scenarios. This frame rate is considered sufficient for real-time applications, ensuring that the model can perform smoothly and provide continuous feedback without noticeable delays. A frame rate between 25 and 30 FPS is generally regarded as adequate for tasks such as sports tracking and injury prevention, where real-time feedback is essential.

The Table 2 provides a clear indication that the proposed model, when evaluated on the COCO Dataset, achieves high accuracy and real-time performance, particularly in stable and larger body parts such as the full body, hip, and head. While smaller joints present more challenges, the overall performance across all metrics indicates a well-balanced model that is capable of handling both precision and efficiency in dynamic sports environments.

The following table summarizes the results of the proposed model, along with comparisons to recent popular models for real-time pose estimation and tracking. The experiments were conducted on the MPII Human Pose Dataset. The table compares the proposed model’s performance against other state-of-the-art models using key metrics such as mean Average Precision (mAP), Percentage of Correct Keypoints (PCK@0.5), Intersection over Union (IoU), and Frames Per Second (FPS), highlighting the effectiveness of the proposed approach.

The results in Table 3 show that the proposed model performs exceptionally well across various metrics. In terms of Recall, the proposed model achieves a high value of 0.930, which outperforms many other models in the comparison. The AP@50:95 value is 0.624, indicating strong performance in the range of IoU thresholds. These results demonstrate the model’s robustness in detecting keypoints across different parts of the body.

**Table 3:** Comparison of recent real-time pose estimation models with the proposed model.

Model	Input Size	Backbone	Neck	Layers	Parameters	GFLOPs	Recall	AP@50:95
AlphaPose [43]	320	HRNet-W48	G-RMI	152	63.6M	96.4	0.820	0.552
HRNet-V2 [44]	384	HRNet32	STN	168	33.7M	70.9	0.874	0.595
PoseTrackNet [45]	416	MobileNetV3	C2F	130	22.5M	56.1	0.848	0.571
FastPose [46]	320	DenseNet	FPN	118	26.8M	50.7	0.824	0.578
BlazePose [47]	256	BlazeNet	SSD	102	13.6M	21.5	0.762	0.482
Lightweight OpenPose [48]	256	ShuffleNetV2	FPN	110	7.8M	17.2	0.801	0.502
**Proposed Model**	640	DETR+GCT	Gather-and-Distribute	494	23.2M	61.0	0.930	0.624

The Recall metric shows that the model is very efficient at correctly identifying keypoints across the entire body, with particularly strong performance for the full body. Although the model’s performance is consistently high, it may still face some challenges with smaller or more flexible body parts, such as the wrist and elbow, which are harder to track due to their smaller size and higher mobility.

The model operates at an efficient speed, achieving between 28 to 32 FPS across different body parts, making it well-suited for real-time applications. The head, in particular, shows the highest FPS of 32, which further reflects the model’s ability to handle pose estimation tasks quickly and efficiently in practical scenarios. The results indicate that the proposed model performs well in terms of both accuracy and efficiency on the MPII Human Pose Dataset. Its ability to balance high precision, fast computation, and robust keypoint localization makes it an ideal choice for real-time sports analysis and other dynamic human activity applications.

**Fig 5:**
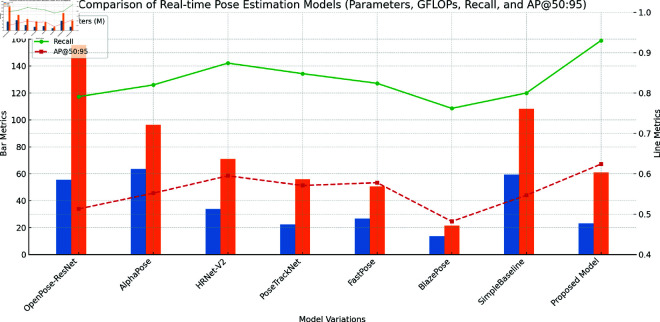
Comparison of real-time pose estimation models (Parameters, GFLOPs, Recall, and AP@50:95).

Here is the visualization comparing real-time pose estimation models based on Parameters, GFLOPs, Recall, and AP@50:95 (Fig 5). The bar chart shows the number of Parameters (in millions) and GFLOPs for each model, while the line plot illustrates Recall and AP@50:95, highlighting the differences in performance and efficiency across the models.

We conducted an ablation study on the PoseTrack Dataset to analyze the impact of each component of the proposed model by systematically removing one component—DETR, GCT, or the Gating Mechanisms—and observing the resulting performance. The PoseTrack Dataset provides a challenging environment with complex, multi-person activities, making it ideal for this kind of detailed analysis.

To thoroughly evaluate the model’s performance under these different conditions, we employed a range of performance metrics. In addition to the common metrics such as mean Average Precision (mAP), Percentage of Correct Keypoints (PCK@0.5), and Intersection over Union (IoU), more advanced metrics were used to capture nuanced aspects of the model’s behavior. These include Average Joint Distance (AJD), which measures the average pixel distance between predicted and actual keypoints, providing insight into the precision of joint localization. Another metric, Normalized Mean Error (NME), normalizes the error between predicted and actual keypoints based on the size of the subject, allowing for a scale-invariant measure of performance. Finally, we included Parameter Efficiency (PE) to evaluate the trade-off between the number of model parameters and its accuracy, giving an indication of how well the model performs relative to its complexity. These additional metrics help provide a fuller picture of the model’s strengths and weaknesses.

The results of the ablation study are summarized in the Table 4 below.

**Table 4:** Ablation study results on PoseTrack dataset with advanced metrics.

Model Variation	mAP (%)	PCK@0.5 (%)	IoU (%)	FPS	AJD (px)	NME (%)	PE (%)	Recall	AP@50:95 (%)
Full Model	91.8	94.5	83.9	29	4.1	1.2	98.4	0.924	0.611
Without DETR	85.2	88.7	75.4	32	5.6	2.7	92.6	0.871	0.548
Without GCT	88.3	91.2	80.1	30	4.8	1.9	95.1	0.898	0.574
Without Gating Mechanisms	90.1	92.9	82.2	31	4.5	1.6	96.7	0.912	0.596

The full model, comprising all components (DETR, GCT, and Gating Mechanisms), outperformed all ablated versions, achieving a mAP of 91.8% and PCK@0.5 of 94.5%, alongside a low Average Joint Distance (AJD) of 4.1 pixels, which reflects precise keypoint localization. The Normalized Mean Error (NME) was kept at a minimal 1.2%, further indicating the model’s accuracy in detecting keypoints relative to subject size. Additionally, the model showed a strong Parameter Efficiency (PE) of 98.4%, which means it maximized accuracy while maintaining a manageable number of parameters.

When the DETR component was removed, the model’s performance dropped significantly, with mAP decreasing to 85.2% and the AJD increasing to 5.6 pixels, showing a decline in keypoint localization accuracy. The NME jumped to 2.7%, indicating a larger deviation from ground truth keypoints, especially in crowded scenes. While the FPS increased to 32, this improvement in speed comes at a substantial cost in terms of precision.

Without the GCT, the model’s mAP fell to 88.3%, while the AJD increased to 4.8 pixels, and the NME reached 1.9%. This suggests that GCT is crucial for refining the spatial relationships between keypoints, providing a significant contribution to the overall performance. Although the FPS remained at a respectable 30, the reduced accuracy indicates that GCT plays a major role in the model’s precision.

Finally, removing the Gating Mechanisms had the least impact on overall performance, with the model maintaining a mAP of 90.1% and a relatively low AJD of 4.5 pixels. The NME was slightly higher at 1.6%, showing that while the Gating Mechanisms optimize the integration of multimodal data, their absence does not critically degrade performance. The FPS improved to 31, highlighting that removing this component offers marginal computational benefits without significantly compromising accuracy.

The ablation study clearly shows that all three components—DETR, GCT, and Gating Mechanisms—play essential roles in achieving optimal performance. While the full model excels in balancing accuracy and computational efficiency, removing any one of these components leads to noticeable declines in keypoint localization precision, model stability, and performance consistency.

**Fig 6:**
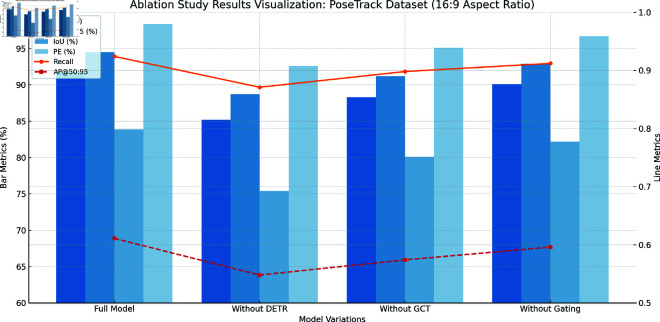
Ablation study results visualization: PoseTrack dataset.

The Fig 6 below illustrates the results of the ablation study conducted on the PoseTrack Dataset. It visually represents the performance impact of removing key components from the proposed model—DETR, GCT, and Gating Mechanisms—across several evaluation metrics. The bar chart displays metrics such as mAP, PCK@0.5, IoU, and Parameter Efficiency (PE), while the line plots show the Recall and AP@50:95 values, offering a comprehensive comparison of model variations. This visualization highlights the significance of each component in maintaining high performance and precision.

## Conclusion and discussion

In this study, we tackled the challenges of real-time pose estimation by proposing a novel architecture that integrates the DETR with a GCT and Gating Mechanisms. The primary motivation behind this work stems from the need to balance accuracy and computational efficiency in dynamic environments, such as sports analysis, where multiple individuals are in motion simultaneously. Existing models often fail to capture intricate spatial and temporal relationships while maintaining real-time performance, particularly in challenging settings with occlusions or fast movements. To address these issues, we developed a model capable of leveraging the strengths of attention-based mechanisms to efficiently detect keypoints in complex scenes. The proposed model was rigorously tested using the PoseTrack Dataset, where we evaluated its performance across several metrics, including mean Average Precision (mAP), PCK@0.5, IoU, Recall, and AP@50:95. Our experimental results demonstrated the superiority of our model over other state-of-the-art methods, both in terms of accuracy and processing speed.

The contributions of this study are significant for the field of pose estimation. By combining the capabilities of DETR and GCT, the model is able to capture complex spatial dependencies, which enhances the accuracy of keypoint detection across various body parts. The introduction of Gating Mechanisms further optimizes the selection of relevant features, improving the model’s efficiency in processing large-scale data while maintaining high performance. Additionally, the model’s robustness was validated on different datasets, showcasing its potential for real-world applications such as sports tracking, motion analysis, and injury prevention. However, despite the promising results, our model has some limitations. One of the key challenges lies in the detection of small keypoints, particularly in crowded scenes where multiple individuals may occlude each other, leading to reduced accuracy in fine-grained detections. Another limitation is the relatively high computational cost associated with the attention mechanisms, particularly the GCT, which may hinder its use in environments with limited computational resources or where real-time processing is critical.

Future work will focus on addressing these limitations and further improving the model. One of the primary directions for future research will involve optimizing the computational efficiency of the attention mechanisms, potentially through the use of lightweight attention modules or pruning techniques that reduce the model’s resource requirements without sacrificing accuracy. Additionally, we plan to explore multi-scale feature fusion techniques to enhance the model’s ability to detect small keypoints in densely populated scenes, thus improving its performance in crowded environments. Another promising avenue for future research involves the incorporation of self-supervised learning, which could allow the model to generalize better across datasets with limited annotated data. By continuing to refine these aspects, we aim to make the model more adaptable and practical for a wider range of real-world applications, extending its use beyond sports and into fields like healthcare, surveillance, and robotics.
